# Immunogenicity and Blocking Efficacy of Norovirus GII.4 Recombinant P Protein Vaccine

**DOI:** 10.3390/vaccines11061053

**Published:** 2023-06-01

**Authors:** Zhendi Yu, Qingyi Shao, Zhangkai Xu, Chenghao Chen, Mingfan Li, Yi Jiang, Dongqing Cheng

**Affiliations:** School of Medical Technology and Information Engineering, Zhejiang Chinese Medical University, Hangzhou 310053, China; 202111116011021@zcmu.edu.cn (Z.Y.);

**Keywords:** norovirus, recombinant P protein, neutralizing antibody, immunogenicity, blocking efficacy

## Abstract

Noroviruses (NoVs) are the main cause of acute gastroenteritis in all ages worldwide. The aim of this study was to produce the recombinant P protein of norovirus and to demonstrate its blocking effect. In this study, the engineered strains were induced to express the P protein of NoVs GII.4, which was identified using SDS-PAGE and ELISA as having the capacity to bind to histo-blood group antigens (HBGAs). Rabbits were immunized to obtain neutralizing antibodies. ELISA and ISC-RT-qPCR were used to determine the blocking efficacy of the neutralizing antibody to human norovirus (HuNoV) and murine norovirus (MNV). The recombinant P protein (35 KD) was obtained, and the neutralizing antibody was successfully prepared. The neutralizing antibody could block the binding of the P protein and HuNoV to HBGAs. Neutralizing antibodies can also block MNV invasion into host cells RAW264.7. The recombinant P protein expressed in *E. coli* can induce antibodies to block HuNoV and MNV. The recombinant P protein of NoVs GII.4 has the value of vaccine development.

## 1. Introduction

Noroviruses (NoVs) are one of the main pathogens that cause non-bacterial acute gastroenteritis in humans, and it is also the pathogen of the most serious food-borne infectious diseases. About 90% of non-bacterial diarrhea is caused by NoVs [1]. NoVs are mainly transmitted via the fecal-oral route and have a high incidence in winter. NoVs are highly resistant to the environment and can survive on surfaces for up to two weeks and in water for over two months. A low infectious load of the human norovirus (HuNoV) infection comprises a minimum of 18 viral particles that cause infection [2]. Headache, abdominal pain, nausea, emesis, and other symptoms occurr after 24–48 h in infected patients [3]. The infection is self-limited in people with normal immunity, but it takes a long time to recover in children under 5 years of age, people over 65 years of age, and patients with immunodeficiency. It can cause severe disease, leading to dehydration, shock, and even death [1]. Outbreaks of foodborne and waterborne NoV infections have continued to increase in recent years [4]. Therefore, it is necessary and urgent to strengthen research on vaccines against NoVs.

As a result of a lack of effective drugs and treatment, the promotion and use of vaccines are the most effective way to control NoV infections [5]. There are many factors in the establishment of experimental models, such as high experimental requirements, complex procedures, adverse reactions to vaccines, and safety in clinical trials, which hinder the development and promotion of vaccines [6]. Recently, it has been found that HuNoV can be cultured in B cells and human intestinal enteroid. However, as a result of the low replication efficiency of the virus, this technology is not widely used for vaccine development [7]. As a result of these limitations, no NoV vaccines have been licensed worldwide. Recombinant HuNoV capsid proteins, expressed either by eukaryotic or prokaryotic systems, are often used in studies on immunogenicity, diagnosis assays, and host–receptor interactions [8,9].

This study aimed to express and obtain the NoV recombinant P protein, prepare an NoV neutralizing antibody using the P protein as an antigen, and further study the protective efficacy of the recombinant P protein neutralizing antibody against NoVs. This was undertaken to explore the potential for developing NoV component vaccines using recombinant P protein as an antigen.

## 2. Materials and Methods

### 2.1. Viruses, Cells, and Experimental Animals

The recombinant P protein of NoVs was expressed using an engineered bacterium, pET28a-inaQn-TB-P(GII.4)/BL21, following a previous report [10] provided by Dr. Wang Dapeng from Shanghai Jiaotong University. Clean-grade rabbits were obtained from the Animal Experimental Research Centre of the Zhejiang Chinese Medical University. The animal ethics approval number was IACUC-20211108-17. Murine norovirus (MNV) and RAW264.7 cells were kept in a laboratory.

### 2.2. Expression and Purification of Recombinant P Protein of NoVs

The recombinant P protein of NoVs was expressed in a prokaryotic expression host. The activated bacterial culture was induced with an isopropyl β-D-1-thiogalactopyranoside (IPTG; Sangon Biotech, Shanghai, China) solution (final concentration 0.5 mmol/L) and grown in an LB medium (Sangon Biotech, Shanghai, China) containing 100 μg/mL of kanamycin. The bacterial culture was harvested and treated with bovine thrombin (effective cleavage ratio of 1:2000) at 37 °C for 3 h. The supernatant after enzyme digestion was purified using Ni-IDA affinity chromatography (Sangon Biotech, Shanghai, China). The purified protein was then identified using SDS-PAGE (10% separating gel and 5% concentrating gel) and confirmed to be 35 KD in size. The protein concentration was determined using Nanodrop (Thermo, Waltham, MA, USA).

### 2.3. Determining the Receptor Binding Capacity of Recombinant P Protein Using Indirect ELISA

Following a previously published protocol [11], the recombinant P protein was coated on an ELISA plate overnight at 4 °C. After blocking with 1% BSA at 37 °C for 1 h, the plate was washed with PBS three times, and HBGAs were added to test wells, while PBS was used as a negative control. The plate was incubated at 37 °C for 1 h with three replicates, followed by washing to remove unbound material. Then, HBGA monoclonal antibodies (MAbs, diluted to 1:1000 with PBS containing 5% BSA; Covance, Princeton, NJ, USA) were added to all wells and incubated at 37 °C for 1 h. Type A and H HBGA monoclonal antibodies were IgG antibodies, and type B HBGA monoclonal antibodies were IgM antibodies. After washing, secondary antibodies HRP-conjugated goat anti-mouse IgG (HCL chains, diluted to 1:10,000 with PBS containing 5% BSA; Yeasen, Shanghai, China) and HRP-conjugated goat anti-mouse IgM (HCL chains, diluted to 1:10,000 with PBS containing 5% BSA; Yeasen, Shanghai, China) were added and incubated at 37 °C for 1 h. HRP-conjugated goat anti-mouse IgG was used for type A, and H HBGA monoclonal antibodies and HRP-conjugated goat anti-mouse IgM was used for type B HBGA monoclonal antibodies. A TMB substrate was added, and the plate was incubated at 37 °C for 10 min for color development. The reaction was stopped with H_2_SO_4_. The absorbance per well at 450 nm was detected using a microplate reader (Tecan, Männedorf, Switzerland). A sample was considered positive for binding to HBGAs if the absorbance value of the sample was 2.1-fold higher than that of the negative control.

### 2.4. Production and Determination of Neutralizing Antibodies to NoV Recombinant P Protein

Freund’s incomplete adjuvant (FIA) was prepared by mixing lanolin with liquid paraffin at a ratio of 1:3. Instructions for our preparation process are as follows: Preheat FIA at 60 °C for 30 min, absorb 3 mL into a mortar, and add 0.5 mL of BCG and 2.5 mL of a 2.4 mg/mL NoV recombinant P protein solution drop by drop. Grind until a homogeneous emulsion is formed, and if 1 drop of the emulsion can be placed on cold water without dispersing, the solution is counted as qualified, indicating that the adjuvant has formed a water-in-oil state with the antigen for immunization. The NoV recombinant P protein was made into a water-in-oil immune emulsion (Freund’s complete adjuvant was used for the primary immunization, and Freund’s incomplete adjuvant was used for the booster immunization). Eight rabbits were randomly divided into the experimental group and the negative control group. Each rabbit of the experimental group was injected with the antigen emulsified with Freund’s complete adjuvant via a syringe at 8 points on both sides of the rabbit spine. A total of 0.2 mL of the antigen was injected at each point, and the booster immunization was given in intervals of one week a total of three times. Neutralizing antibodies were collected from the carotid artery after the last immunization.

The titer of the antibody was detected using a double immunodiffusion test. Gel plates were prepared by pouring heated, melting 1% agarose. P protein was added to the central wells, and 1:2, 1:4, 1:8, 1:16, and 1:32 diluted antibodies were added to the peripheral wells. The plates were incubated overnight at 37 °C in a wet box and observed the next day for the appearance of precipitation lines. The highest serum dilution with a precipitation line was regarded as the titer of the polyclonal antibody.

### 2.5. Detecting Blocking Efficacy of Neutralizing Antibody

A total of 100 μL of a 50 μg/mL NoV recombinant P protein was coated overnight at 4 °C. The P protein was dissolved in a 0.05 mol/L pH9.6 carbonate-coated buffer solution and blocked using a 1% BSA at 37 °C for 1 h. After washing, a 1:2 dilution of the neutralizing antibody was added for the blocking test. A control group serum was used as a non-blocking control and incubated at 37 °C for 1 h. HBGAs were added and incubated for 1 h at 37 °C. After washing, the HBGA monoclonal antibody was added and incubated. HRP-conjugated goat anti-mouse IgG and HRP-conjugated goat anti-mouse IgM were added as secondary antibodies. After incubation, TMB and H_2_SO_4_ were added, and the absorbance *A* value was read at 450 nm.

Blocking ratio = (*A_control_* − *A_blocking_*)/*A_control_* × 100%. (1)

The blocking effect of the neutralizing antibodies on HuNoV was detected using ISC-RT-qPCR, as described by Peng et al. [12,13]. Viral receptor HBGAs were coated with a NuncTopYield plate (Thermo, Waltham, MA, USA) at 4 °C overnight, blocked with a 1% BSA at 37 °C for 1 h, and washed before use. The P protein neutralizing antibody (1:2 dilution) was mixed with HuNoV and incubated at 37 °C for 1 h as the blocking experimental group. The control group serum was used as the unblocked control group. The plate was incubated at 37 °C for 1 h and washed to remove uncaptured viruses. RNase-free water was added to each well, and then the mixture was cooled at 4 °C for 5 min after a metal bath at 95 °C. A viral nucleic acid was released. The viral load was detected using a reverse transcription quantitative real-time PCR assay (RT-qPCR).

The viral RNA was used as a template for RT-qPCR detection with the One Step Primescript RT-PCR Kit (Takara, CA, Shiga, Japan). Information on the primers and probes used is shown in Table 1. Each 25 μL PCR reaction included a 12.5 μL 2 × PCR buffer, a 0.5 μL Takara Ex Taq HS, a 0.5 μL RT enzyme, a 0.6 μL forward primer and reverse primer, a 0.3 μL probe, a 5 μL RNase-free ddH_2_O, and a 5 μL viral RNA template. The RT-qPCR cycling profile used was set at 42 °C for 30 min, 95 °C for 2 min, 95 °C for 5 s, and 55 °C for 35 s. There were 40 cycles.

The recombinant P protein neutralizing antibody was diluted to titers of 1:2, 1:4, 1:8, 1:16, and 1:32. A 500μL diluted neutralizing antibody was mixed with an equal MNV solution and incubated for 1 h at 37 °C with PBS as an unblocking control. The different mixtures were added to the corresponding plates with RAW264.7 cells. The cells without viruses were used as normal cell control and were cultured at 37 °C for 2 h. The mixture was discarded and washed three times with PBS, and a 2 mL cell maintenance solution was added. The cells were cultured at 37 °C in 5% CO_2_. The cells were observed daily, their cytopathic effects were recorded, and they were repeatedly freeze-thawed to release the viruses. NoV RNA was extracted and detected using RT-qPCR. The viral load in the neutralizing antibody blocking group was significantly less than that of the non-blocking group, indicating that it had a blocking effect [14,15].

### 2.6. Statistical Analysis

Triplicate measurements were performed as biological replicates for each experiment. The significance of the variation between groups was examined using a *t*-test, while a *p*-value < 0.05 was considered a statistically significant variation between groups. All the data were analyzed using SPSS 23.0.

## 3. Results

### 3.1. Expression and Purification of Recombinant P Protein

**Figure 1:**
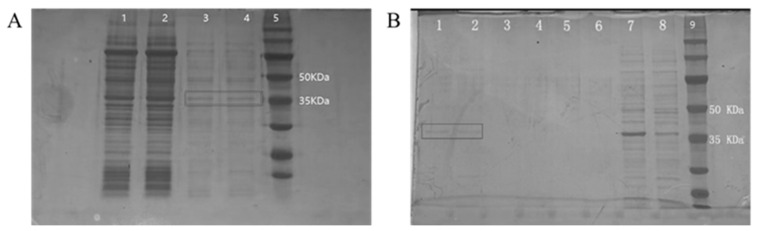
SDS-PAGE results of NoV recombinant P protein. (**A**) Lanes 1 and 2 are bacterial proteins, lanes 3 and 4 are the enzymatic supernatant, and lane 5 is the protein marker. (**B**) Lanes 1, 2, and 3 are eluents 1,2, and 3 through the Ni column, and lanes 4, 5, and 6 flow through the liquid. Lines 7 and 8 are bacterial proteins. Line 9 is the protein marker.

The NoV recombinant P protein was induced using IPTG, and an enzyme digestion supernatant was obtained. The results of SDS-PAGE indicate a clear band at 35 KDa when compared with the protein marker (Figure 1A). This is consistent with expectations, indicating that the digestion was successful and a supernatant containing the NoV recombinant P protein was obtained, yet there were miscellaneous proteins in the supernatant.

The supernatant of enzyme digestion was separated and purified using the histidine tag protein purification method. According to SDS-PAGE detection (Figure 1B), the NoV recombinant P protein was mainly present in eluates 1 and 2, and there were a few miscellaneous proteins. Eluate 3 and flow solutions 1, 2, and 3 did not contain the recombinant P protein. Further, the recombinant P protein was 35 KDa, meaning it could be used for the subsequent preparation of neutralizing antibodies.

### 3.2. Binding Capacity of Recombinant P Protein to HBGAs

**Figure 2:**
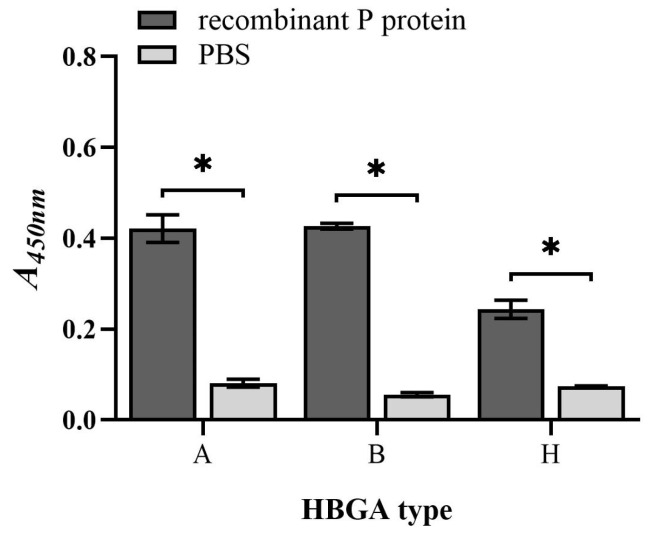
ELISA detected the binding capacity of recombinant P protein to HBGAs. “*” indicates *p* value < 0.05.

Indirect ELISA was used to verify the binding capacity of the recombinant P protein to HBGAs. The experimental results show that the purified recombinant P protein of NoVs could bind to types A, B, and H HBGAs (Figure 2). The absorbances of the recombinant P protein to type A, type B and type H HBGAs were 0.42, 0.43, and 0.24, respectively, which is significantly higher than those of the PBS group (*p* < 0.001). Meanwhile, the S/N ratios were all greater than 2.1 [11]. The results confirm that the recombinant P protein of NoV-induced expression had the ability to bind to viral receptor HBGAs. P protein can replace NoVs in subsequent experiments, such as the preparation and blocking of neutralizing antibodies.

### 3.3. Neutralizing Antibody Preparation and Titer Determination

After primary immunization with a water-in-oil emulsion made from the NoV recombinant P protein, three-booster immunization was performed. Blood samples were collected from the carotid artery, and serum samples were separated. Neutralizing antibodies were detected using the a double immunodiffusion test. The titers of the antibodies were 1:32, 1:16, 1:16, and 1:8. A neutralizing antibody against the NoV recombinant P protein was successfully prepared.

### 3.4. Blocking Efficacy of Neutralizing Antibody to HuNoV

**Figure 3:**
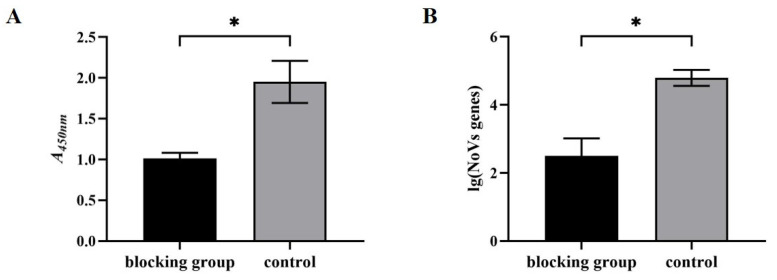
Blocking effect of the neutralizing antibody to HuNoV. Neutralizing antibodies blocked (**A**) the binding of recombinant P protein to HBGAs and (**B**) the binding of HuNoV to HBGAs. “*” indicates *p* value < 0.05.

Saliva was used as the NoV receptor HBGAs [16], the ELISA method was used to analyze the blocking effect of neutralizing antibodies on the binding of the P protein to the viral receptor. The control group serum was used as an unblocked control. The blocking rates were 41.99% and 48.02%. The average blocking rate was 45.00% (Figure 3A). The difference in average absorbances was significant (*p* < 0.05). It was preliminarily confirmed that the neutralizing antibody could block the binding of the P protein to HBGAs. The P protein is a possible binding site for NoV infection. The blocking effects on the P protein and HBGAs suggests that the antibody may be effective against HuNoV and MNV particles.

The antibodies were then used to block the binding of HuNoV to HBGAs. Since there is no mature cell culture system for HuNoV, the ISC-RT-qPCR method was used for detection. The HuNoV was treated with the neutralizing antibody. The viral load of the antibody treatment group was 2.50 log 10, which was significantly lower than that of the control group, 4.79 log 10 (Figure 3B). As a result of the blocking of neutralizing antibodies from the HuNoV surface P protein, the viruses were unable to bind to HBGAs, thereby reducing the viral load. This indicates that the neutralizing antibody had a certain blocking efficacy on the binding of HuNoV to the receptor HBGAs.

### 3.5. Blocking Efficacy of Neutralizing Antibody to MNV Invasion

**Figure 4:**
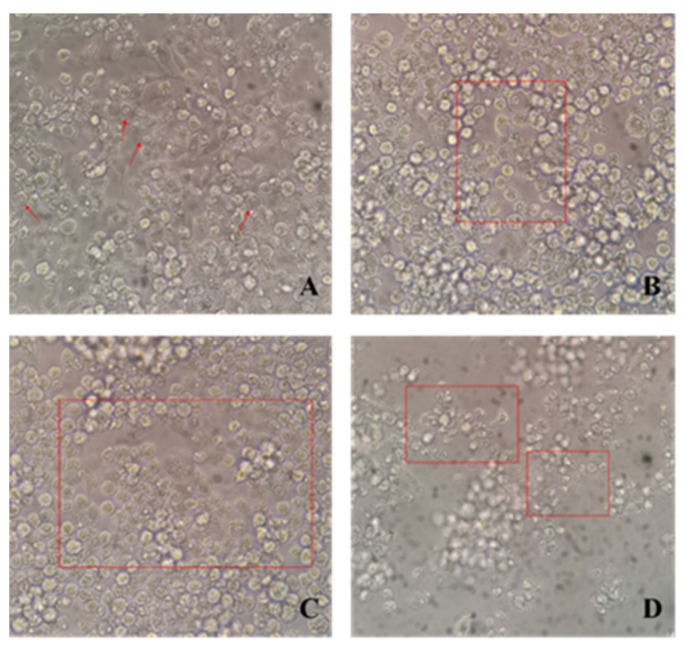
Cytopathic effects of MNV infection in RAW264.7 cells. (**A**) Vacuoles and nuclear pyknosis, (**B**) infection of nearby cells, (**C**) a large amount of cell nuclear pyknosis and cell death, and (**D**) cell shrinkage and fragmentation. Red arrows indicate the vacuoles of cells. Red frames indicate the sites of cytopathic effects.

MNV is often used as an alternative to HuNoV in vitro. Therefore, the blocking efficacy of neutralizing antibodies on MNV invasion was determined. Gradient-diluted neutralizing antibodies were incubated with MNV, and RAW264.7 cells were inoculated into a monolayer. A cytopathic effect was clearly observed. The infected cells showed vacuoles and nuclear pyknosis (Figure 4A), and surrounding cells were invaded afterwards (Figure 4B). Subsequently, a large number of cell nuclei were pyknotic and aggregated, some cells died, and cavities appeared (Figure 4C). Finally, massive cells died, and the adherent cells shrank and fragmented (Figure 4D).

**Figure 5:**
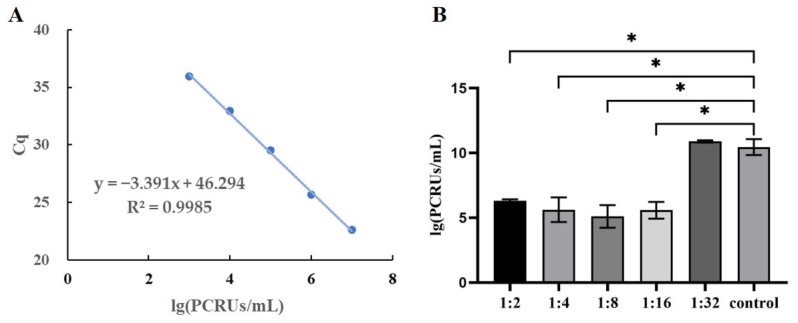
Viral loading in RAW264.7 after blocking with neutralizing antibody. (**A**) Standard curve of MNV viral loading. (**B**) Protective efficacies of neutralizing antibodies with different titers. “*” indicates *p* value < 0.05.

The cells were subsequently lysed. The viruses were released, and the viral load was measured using RT-qPCR. When the dilution of the NoV neutralizing antibody was less than 1:16, the amount of virus in the blocking group (5.57 log 10) was significantly lower than that in the non-blocking group (10.45 log 10), and the difference was statistically significant (*p* < 0.0001, Figure 5). This means that the virus surface P protein was blocked by neutralizing antibodies during incubation with the virus, so the host cells avoided invasion by the virus. However, when the antibody was diluted to 1:32, the viral load released (10.89 log 10) was not significantly different from that of the control group (*p* = 0.9455). The neutralizing antibodies could block the RAW264.7 cells infected by MNV.

## 4. Discussion

NoVs are highly infectious, with low infective doses and strong environmental resistance. NoVs can easily cause a fulminant epidemic in hospitals, schools, and other gathering populations [17]. At present, there is no specific antiviral drug, and the clinical treatment is mainly symptomatic or supportive [18]. The improvement of sanitary conditions cannot prevent infection by NoVs [19]. The vaccine is the only means to control the epidemic of NoVs [20]. There has been an independent positive correlation found between norovirus and gastroenteritis [21]. NoVs have caused a significant economic burden on world health. The outbreak of COVID-19 has reduced the incidence of norovirus, and some reports predict that the incidence of NoVs may suddenly increase after the outbreak of COVID-19 [22,23]. Therefore, it is necessary to develop vaccines or drugs against this disease as soon as possible. However, up to now, a stable and effective in vitro proliferation model of HuNoV has not been established, and it is impossible to carry out research on inactivated HuNoV vaccine and live attenuated HuNoV vaccine [4].

The whole genome of NoV is about 7.5–7.7 kb and contains three open reading frames (ORFs). ORF1 is related to replication, and ORF2 and ORF3 encode virus proteins (VPs) [24,25]. The NoV capsid protein is composed of VP1 and VP2. VP1 can be divided into a shell domain (S region) and a protruding domain (P region). The P region may be a key region for NoVs to bind to receptors [26,27]. GII.4 is recognized as binding to HBGAs through the P domain of the capsid [28]. Each P region contains three loops as sites of foreign antigen presentation, which can accommodate the insertion of large gene fragments [29]. The P protein is expressed in the P region. Recent studies have shown that the P protein is obtained from prokaryotic expression systems, and that P particles have good immunogenicity. The P particle is not only a good carrier for presenting foreign antigens, but it is also a hot spot in research on NoV subunit vaccines [30,31].

The development of norovirus vaccines faces great difficulties as a result of rapid mutation of the norovirus, a lack of suitable cells for NoV cultures, a lack of effective animal models of infection, and a lack of knowledge about the interaction between NoV and host. The development direction of NoV vaccines is mainly moving toward the P particle vaccine, virus-like particle vaccines, and adenovirus vector vaccines. Currently, there are no approved vaccines or drugs for the treatment of HuNoV infections on the market. In clinical trials on the GI.1 and GII.4 bivalent virus-like particle vaccines, data statistics show that the vaccine is well tolerated [32]. Adenovirus-based GI.1 VP1, developed by Vaxart, was able to induce effective immune responses in mice using intranasal inoculation. The P particle vaccine is another research hotspot after the VLP vaccine. However, its immunogenicity is lower than that of the VLP vaccine, and it needs to be combined with an adjuvant to produce stronger protective effects. A large amount of preclinical vaccine data on GII.4P have been reported [33]. P particles develop similarly to the P domain of norovirus. The P domain is one of the binding sites of HBGA-bound viruses [33] P proteins can also be mass produced in a laboratory and are expressed by Escherichia coli. The use of P proteins could potentially reduce the cost of vaccines [26]. As a result of limitations due to the lack of an in vitro culture system for HuNoV, attenuated and inactivated vaccines against NoVs have not been developed. GII.4 has been the predominant genotype to cause outbreaks worldwide [24]. In this study, a GII.4 NoV P protein was expressed in E. coli and showed binding capacity with HBGAs. As an important receptor or co-receptor of NoVs, HBGAs play an important role in host susceptibility and viral epidemics [34]. HBGAs are complex carbohydrates, which are widely distributed in the mucosal epithelial cells of the respiratory tract and intestine. HBGAs also exist in saliva and other bodily fluids in the form of free oligosaccharide [35]. Different NoV genotypes may bind to different types of HBGAs [22]. GII.4 and GII.17 have been found to interact with all A, B, O, and Lewis type HBGAs [36]. GII.5 and GII.12 can bind to types A and B, but not to O-secretory forms [37]. The importance of an interaction between NoVs and HBGAs was confirmed for host susceptibility and immune resistance. HBGAs can act as a receptor for NoVs and are a sensitive factor for hosts of NoVs [38]. NoV-specific antibodies can block the binding of NoV VLPs to HBGA receptors or attachment factors. Scholars have recognized that serum HBGAs that block antibodies can be used as a surrogate indicator of neutralizing antibodies [39].

A study on human volunteers showed that levels of functional antibodies in the volunteers’ serum prevented the binding of NoV VLPs to HBGAs. Therefore, antibody levels have become an indicator for evaluating the immune protective ability of NoV vaccines [19,40]. This is the same conclusion as was found from our experimental results. One study collected saliva samples up to day 84 of infection and serum samples from acute and convalescent norovirus-infected patients. The results showed that salivary IgA levels were closely related to an increase in IgA titer and blocking antibodies in the convalescent serum. This suggests that mucosal IgA is a key factor in protecting a host from NoV infection [41,42]. In addition to this, several clinical studies have shown that serum IgA, memory B cell response, and serum HBGA blocking titer are also associated with immune protection [43,44].

Adaptive immune responses include humoral immunity and cellular immunity. In this study, only the serum antibody level after immunization with the P protein was analyzed, and the effects of different doses or types of adjuvants and immunization methods on immunogenicity need to be further studied. However, the level of cellular immunity was not evaluated in this study, so the next step is to sort different types of T cells and B cells to provide more data support for the effects of cellular immunity. In addition, the pathogenic mechanism of NoVs and their process of infecting cells are still unclear, and a mechanistic study of NoVs could further provide strong support for the development of NoV vaccines or antiviral drugs.

The NoV neutralizing antibody prepared in this paper exhibited prominent blocking efficacy against HuNoV binding to HBGAs, and MNV binding to host cells. This proves that the recombinant P protein has the potential to be developed into an NoV subunit vaccine. Compared with VLP, P protein has a lower molecular weight. P protein is expressed easily and at a low cost, which is more valuable for development. However, the blocking efficacy analysis in this study was based on in vitro experiments. It is necessary to verify the protective effects of neutralizing antibodies against HuNoV on the host in animal experiments. In this study, serum antibody levels after P protein immunization were analyzed, but the indicators of cellular immunity were not further developed, which also needs further proof. These results lay the foundation for the subsequent development of recombinant P protein vaccines.

## 5. Conclusions

In this study, a recombinant P protein was produced by *E. coli* containing pET28a-inaQn-TB-P (GII.4). The recombinant P protein, about 35 KDa, was obtained after purification. It has the ability to bind to viral receptor HBGAs. The purified P protein produced a good immune response after immunizing rabbits. Neutralizing antibodies had a certain blocking effect on HuNoV and MNV. It suggests that the recombinant P protein of NoV GII.4 has value in vaccine development.

## Figures and Tables

**Table 1:** The sequence of primers and probes of HuNoV and MNV.

Primer	Sequence (5′-3′)
HuNoV-F	CAAGAGTCAATGTTTAGGTGGATGAG
HuNoV-R	TCGACGCCATCTTCATTCACA
HuNoV-P	FAM-AGATTGCGATCGCCCTCCCA-TAMRAR
MNV-F	CCGCCATGGTCCTGGAGAATG
MNV-R	GCACAACGGCACTACCAATCTTG
MNV-P	RoX-CGTCGTCGCCTCGGTCCTTGTCAA-BHQ2

## Data Availability

The datasets generated during the current study are available from the corresponding author on reasonable request.
